# A Chemogenetic Approach for the Optical Monitoring of Voltage in Neurons

**DOI:** 10.1002/anie.201812967

**Published:** 2019-01-25

**Authors:** Mayya Sundukova, Efthymia Prifti, Annalisa Bucci, Kseniia Kirillova, Joana Serrao, Luc Reymond, Miwa Umebayashi, Ruud Hovius, Howard Riezman, Kai Johnsson, Paul A. Heppenstall

**Affiliations:** ^1^ Molecular Medicine Partnership Unit (MMPU) 69117 Heidelberg Germany; ^2^ Epigenetics and Neurobiology Unit EMBL Rome via Ramarini 32 Monterotondo Italy; ^3^ Department of Chemical Biology Max Planck Institute for Medical Research 69120 Heidelberg Germany; ^4^ Ecole Polytechnique Federale de Lausanne ISIC National Centre for Competence in Research (NCCR) in Chemical Biology 1015 Lausanne Switzerland; ^5^ University of Geneva Department of Biochemistry, National Centre for Competence in Research (NCCR) in Chemical Biology 1211 Geneva Switzerland

**Keywords:** fluorogenic probes, genetic targeting, membrane potential probes, protein tags, voltage imaging

## Abstract

Optical monitoring of neuronal voltage using fluorescent indicators is a powerful approach for the interrogation of the cellular and molecular logic of the nervous system. Herein, a semisynthetic tethered voltage indicator (STeVI1) based upon nile red is described that displays voltage sensitivity when genetically targeted to neuronal membranes. This environmentally sensitive probe allows for wash‐free imaging and faithfully detects supra‐ and sub‐threshold activity in neurons.

Complementation and substitution of electrophysiology methods with the non‐invasive optical imaging of neuronal activity is a major technological challenge in neuroscience.^1^ Calcium imaging with genetically encoded indicators is widely used to interrogate the connectivity and function of neural circuits at different spatial and temporal resolutions.^2^ However, as a surrogate for underlying electrical activity, calcium imaging has a number of shortcomings. For example, calcium indicators lack the sensitivity to register sub‐threshold activity, and their slow kinetics and the nature of increases in intracellular calcium levels often preclude recording of high‐frequency firing.^3^ Direct readout of neuronal membrane voltage is therefore necessary for imaging sub‐threshold and inhibitory activity, and for investigating fast‐coordinated phenomena.^4^ As such, significant efforts are being invested in developing probes and improving microscopy for optical monitoring of voltage.^5^, ^6^, ^7^, ^8^

Fluorescent indicators for membrane potential can be divided into two groups; synthetic voltage sensitive dyes (VSD),^5^ and genetically encoded voltage indicators (GEVI), the latter of which are based upon voltage‐sensitive proteins, such as opsins, channels, and phosphatases.^9^ Organic VSDs possess excellent photophysical properties and fast kinetics for live imaging. However, their application in vivo is limited by the unspecific staining of tissue, compromising signal‐to‐noise ratio (SNR) and cell identity.^5^ GEVIs provide a valuable alternative as they can be genetically targeted to subsets of cells.^6^, ^8^, ^10^ However, GEVIs often suffer from low brightness, poor photostability, and slow kinetics.^6^, ^8^ They may also localize poorly to the plasma membrane and exhibit cellular toxicity. To circumvent these problems, hybrid voltage indicators have been proposed that combine the superior optical properties of small‐molecule fluorophores with genetically encoded voltage sensors.^11^ Examples include a precursor VSD that is converted to an active membrane‐bound dye by a genetically encoded enzyme,^12^ and click chemistry‐ and enzyme‐mediated ligation of organic fluorophores to rhodopsin to function as FRET donors.^13^, ^14^

Herein, an alternative approach for hybrid voltage sensor design was considered; the localization of a synthetic voltage indicator to cells of interest using genetically encoded protein tags.^15^ We focused on enzyme‐based, small protein tags, such as the self‐modifying enzyme SNAP‐tag,^16^ and transferase‐mediated labeling of the acyl carrier protein (ACP)‐tag,^17^, ^18^ as these technologies allow for rapid, irreversible labeling, and are compatible with in vivo imaging.^19^ For the VSD component, derivatives of Nile Red, an environment‐sensitive (“fluorogenic”) dye that shows fluorescence enhancement upon transition from aqueous to hydrophobic solvent,^20^, ^21^ register membrane potential with high fidelity. The resulting voltage sensor was named semisynthetic tethered voltage indicator 1 (STeVI1).

The voltage sensitivity of the nile‐red‐derivative NR12S (Figure 1 a), a fluorogenic probe which contains a zwitterionic group and hydrocarbon chain, and has been used to monitor lipid order, was initially investigated.^22^, ^23^ NR12S readily labeled the membranes of HEK293T cells with a signal‐to‐noise ratio (SNR) of 10.45±1.3 under wash‐free conditions (Supporting Information, Figures 1 and 4 a). To quantify its voltage sensitivity, a whole cell voltage clamp was used to control membrane potential, and the epi‐fluorescence signal from the membranes simultaneously recorded at an illumination irradiance of 12 mW mm^−2^. Upon application of rectangular depolarizing voltage steps of various magnitudes from a −60 mV holding potential, fluorescence signal decreased linearly in the physiological range of membrane potential (*R* ^2^=0.97, Figure 1 b). Voltage sensitivity expressed as fractional fluorescence change Δ*F*/*F*% (normalized to fluorescence at a holding potential of −60 mV) achieved −5.1±0.4 % per 100 mV (*n*=10 cells). NR12S fluorescence responded to applied voltage steps with a mean rise time *τ*_on_=1.9±0.4 ms and a weighted decay time *τ*_off_=1.9±0.2 ms (the fast component represented >85 % of response).

**Figure 1:**
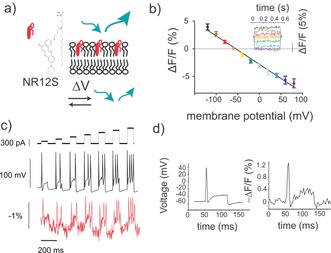
Nile‐red‐based probe NR12S detects membrane voltage change. a) Representation of nile‐red‐based NR12S probe in the membrane. Depolarization of the cell membrane leads to decreased fluorescence intensity. b) Mean response of NR12S to changes in transmembrane potential in patch‐clamped HEK293T cells. Error bars represent 95 % C.I. (*n*=6 cells). The graph inset shows fluorescence responses in a typical NR12S‐labeled HEK293T cell subjected to 500 ms‐long square voltage steps of various magnitudes. Responses were normalized to fluorescence at the −60‐mV holding potential. Line colors match the colors of data points in the main graph. c) Typical NR12S fluorescence response to single‐trial recordings of action potentials in DRG neurons triggered by current injections of incrementing amplitude (80 ms, 80–560 pA, top trace). d) NR12S response to single‐trial recordings of action potentials in DRG neurons triggered by current injection (80 ms, 600 pA). The full width at the half‐maximum of the action potential of the voltage trace (left) was 4.5 ms and of its fluorescence readout was 6.3 ms (right). Images were recorded using an epi‐fluorescent microscope, every 5 ms for (b) and 3 ms for (c,d). Here and subsequently, excitation light was filtered with a bandpass filter (360–540 nm); emission light was filtered with a 570–640 nm filter. Cells were labeled with 500 nm of NR12S for 7 mins at room temperature.

We further enquired whether NR12S could report action potentials in dissociated dorsal root ganglion (DRG) sensory neurons. Imaging at 333 fps allowed for the detection of current injection‐triggered action potentials in single trials, with a Δ*F*/*F*% per action potential of −1.9±0.3 % (*n*=4 neurons), corresponding to a peak SNR of 9.5±1.6 (Figure 1 c,d). Because of the large dynamic range of the probe and its fast kinetics, NR12S fluorescence signals closely followed the action potential shape, permitting the optical monitoring of sub‐ and supra‐threshold neuronal events. Moreover, the orange emission of membrane‐bound NR12S (*λ*_max_=581±1 nm in live cells; Supporting Information, Figure 2), favors this fluorophore over green‐emitting VSDs and GEVIs for use in vivo. Membrane depolarization did not cause a detectable shift in the emission spectrum of NR12S as observed in lipid order monitoring^22^ (Supporting Information, Figure 2 b), therefore the red region of NR12S emission was recorded to detect its fluorescence changes (Figure 1).

We next asked whether nile red derivatives could be genetically localized to cells of interest through binding to a protein tag (Figure 2 a). Nile red derivatives that bind to SNAP‐tag were synthesized and the corresponding tag was expressed on the extracellular surface of HEK293T cells via a glycosylphosphatidylinositol (GPI) anchor signal sequence (Supporting Information, Figure 3 a). Polyethylene glycol (PEG) linkers of *n*=11 repeats (approximately 4.8 nm in length) and charged groups were introduced in the molecules to improve water solubility and reduce nonspecific interactions^24^, ^25^ (Supporting Information, Scheme 1). Although compounds specifically labeled the membranes of cells expressing the SNAP‐tag, negligible voltage sensitivity was observed, as tested by simultaneous patch clamp and imaging (Supporting Information, Figure 3 c,d). Measurements of emission spectra of the compounds from live cells revealed red‐shifted fluorescence in comparison to NR12S (Supporting Information, Table 1), suggesting that compounds were probing hydrophobic surfaces^26^ of the protein tag rather than the membrane environment.

**Figure 2:**
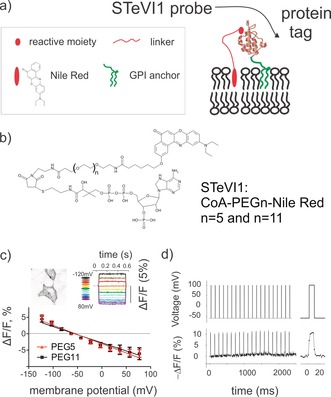
Genetic targeting of nile‐red‐based voltage indicators. a) Illustration of STeVI1–nile‐red derivatives tethered to a protein tag by a molecular linker. b) Structures of ACP‐targeted nile‐red‐derivatives with various linker lengths. c) Mean responses of CoA‐PEG_*n*_‐NR compounds to changes in transmembrane potential in patch‐clamped HEK293T cells, PEG repeats *n*=5, 11. Error bars represent 95 % C.I. (*n*=8–9 cells). The image inset shows representative confocal images of HEK293T cells transfected with ACP‐GPI and labeled with 1 μm CoA‐PEG_11_‐NR. Maximum projection of 19 *z*‐stacks of Δ*z*=0.4 μm. LUT is inverted for illustration purposes. Scale bar 10 μm. The graph inset shows typical fluorescence responses of CoA‐PEG_11_‐NR in a HEK293T cell subjected to a 500 ms‐long square voltage steps of various magnitudes. Responses were normalized to fluorescence at the −60‐mV holding potential. Line colors correspond to different membrane voltages. d) Fluorescence response (bottom) of CoA‐PEG_11_‐NR bound to ACP‐GPI to rectangular voltage steps of 160 mV, applied at approximately 10 Hz (10 ms‐duration, top). Images were recorded using an epi‐fluorescent microscope, every 5 ms for (c) and 2 ms for (d).

To resolve this issue, we reasoned that a smaller tag may give the probe better access to the membrane electric field. The ACP‐tag (8‐kDa) was selected and nile‐red‐Coenzyme A (CoA) conjugates were synthesized with variable PEG repeats (*n*=5, approximately 2.5 nm in length; *n*=11; Figure 2 b; Supporting Information, Scheme 2). The ACP‐tag was expressed on the surface of HEK293T cells via a GPI anchor, and functionality was verified by labeling with CoA‐ATTO532 in the presence of phosphopantetheinyl transferase (SFP‐synthase; Supporting Information, Figure 4 d). Nile red compounds also specifically labeled ACP‐GPI‐expressing HEK293T cells with minimal background (Figure 2 c, Supporting Information, Figures 4 and 5), SNRs of the membrane fluorescence over background under wash‐free conditions were 10.7±1.5 for CoA‐PEG_11_‐NR and 12.4±1.3 for CoA‐PEG_5_‐NR.

ACP‐targeted compounds displayed a significant voltage sensitivity in HEK293T cells which, similar to NR12S, was almost linear (*R* ^2^=0.97 for PEG_11_, *R* ^2^=0.96 for PEG_5_; Figure 2 c and Supporting Information, Movie 1). Fractional fluorescence change per 100 mV was −5.5±0.4 % and −4.9±0.3 % for the CoA‐PEG_5_‐NR and CoA‐PEG_11_‐NR compounds, respectively (*n*=8 cells). This is comparable to the voltage sensitivity of the non‐targetable hemicyanine dye di‐4‐ANEPPS.^27^ The ability of ACP‐tag‐targeted probes to detect trains of action‐potential‐like voltage steps in HEK293T cells was tested (Figure 2 d). The kinetics of the CoA‐PEG_11_‐NR fluorescence response to applied voltage steps was fast, with a mean rise time *τ*_on_=1.8±0.2 ms and a weighted decay time *τ*_off_=2.6±0.1 ms (the fast component represented >85 % of the response).

The voltage sensitivity of CoA‐PEG_*n*_‐NR probes was next investigated in isolated DRG neurons. ACP‐GPI was expressed via recombinant adeno‐associated virus (AAV)‐mediated gene delivery, and functionality was verified by labeling with CoA‐TMR (Supporting Information, Figure 6). STeVI1 compounds selectively labeled the neuronal membranes of the cell body and axons with negligible intracellular signal (Figure 3 a; in part due to charged groups in the CoA; Figure 2 b) and no toxicity. Neurons were patch clamped, and action potentials were evoked via current injection. CoA‐PEG_*n*_‐NR compounds tracked single and trains of action potentials in the cell body (Figure 3 b,c, Supporting Information, Figure 7 and Movie 2). Δ*F*/*F*% amplitudes per action potential reached −2.0±0.3 % for PEG_11_ and −2.2±0.3 % for PEG_5_, respectively (*n*=5 neurons). Corresponding action potential peak SNRs were 13.8±1.5 for PEG_11_ and 16.2±3.0 for PEG_5_. ACP‐GPI expression and probe insertion in the membrane did not cause significant differences in action potential amplitude and duration, or membrane capacitance between control and labeled neurons (One‐Way ANOVA, *p*=0.11, *p*=0.72, and *p*=0.58).

**Figure 3:**
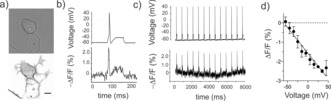
Tethered nile red indicators detect evoked and spontaneous neuronal activity. a) Representative confocal images of cultured DRG neurons expressing ACP‐GPI via rAAV‐mediated delivery. Top, brightfield image, bottom, maximum projection of 34 *z*‐stacks of Δ*z*=0.4 μm. Scale bar 10 μm. b) Typical CoA‐PEG_11_‐NR fluorescence response in a single‐trial recording of an action potential in labeled DRG neurons triggered by current injection (80 ms, 80 pA). The full width at the half‐maximum of the action potential of the voltage trace (top) was 2 ms and of its fluorescence readout was 5 ms (bottom). c) Representative single‐trial recordings of current‐triggered injection (80 ms, 80 pA) action potentials in DRG neurons with 2 μm CoA‐PEG_11_‐NR probe bound to ACP‐GPI. Images were recorded using epi‐fluorescence microscope at 500 fps. d) Fluorescence change vs. membrane potential (mean±standard deviation) displays the linearity of the voltage sensitivity in neurons (data from the trace in (c)). Fluorescence changes corresponding to the membrane voltage binned to 10 mV intervals were averaged and then fitted with a linear function.

Importantly, in neurons nile red fluorescence reported voltage changes linearly (*R* ^2^=0.94, slope=−0.027; Figure 2 d). Fluorescent traces from cell membranes closely mimicked the shape of electrophysiological recorded action potentials, as evidenced by the close match of full width at half‐maximum between the fluorescent and electrical traces (Figure 1 c and b). Indeed, from the voltage imaging, it was possible to discern the inflection on the falling phase of action potentials that is indicative of nociceptive neurons^28^ (see Figure 7 in the Supporting Information for an example). We further investigated whether the nile red probes would allow for the detection of spontaneous activity in neuronal processes. Wide‐field fluorescence imaging of neurons labeled with CoA‐PEG_11_‐NR faithfully reported spontaneous spikes at the level of the cell body and axons in single‐trial optical recordings (Figure 4, Supporting Information, Movie 3). Δ*F*/*F*% amplitude per spontaneous spike at approximately 10 Hz was −2.3±0.1 % at the cell body membrane, corresponding to a peak SNR of 7.6±0.1.

**Figure 4:**
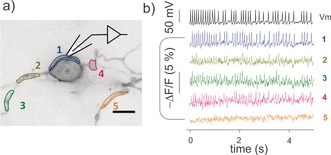
Tethered nile red indicators track spontaneously occurring action potentials. a) Wide‐field image of DRG culture expressing ACP‐GPI and labeled with CoA‐PEG_11_‐NR. Cell body and individual axons are outlined with colored ROIs. Scale bar is 10 μm. LUT image is inverted. b) Membrane potential (top trace, black) was recorded from the cell body under current clamp mode (no current injection). Epi‐fluorescence (measured at 100 fps) from single‐trial optical recordings for the color‐matched somatic and axon areas (colored traces) highlighted in the image. No fluorescence signals were observed for axon area 5 (another neuron).

In conclusion, we demonstrate that nile red derivatives display a voltage sensitivity that can be exploited to monitor membrane potential in genetically tagged cells. Future studies will explore the contribution of possible mechanisms underlying nile red voltage sensitivity, such as solvatochromism, electrochromism, and intermolecular interactions. STeVI1 probes were able to detect the shape of sub‐threshold depolarizations and fast neuronal activity with sensitivity comparable to GEVIs, but required two‐fold less light power (comparison between the data and the references in Table 2 in the Supporting Information). Key to the success of this approach was the small size of the ACP‐tag, which allowed for the positioning of the nile red in the membrane environment. In future applications, this small size may also enable the insertion of the ACP‐tag in exposed loops of channels or other membrane proteins, to direct expression to defined neuronal subcellular compartments, such as axon terminals, soma or dendrites.

## Conflict of interest

The authors declare no conflict of interest.

## Supporting information

As a service to our authors and readers, this journal provides supporting information supplied by the authors. Such materials are peer reviewed and may be re‐organized for online delivery, but are not copy‐edited or typeset. Technical support issues arising from supporting information (other than missing files) should be addressed to the authors.

Supplementary

Supplementary

Supplementary

Supplementary
