# Supplementary material for: A Chemogenetic Approach for the Optical Monitoring of Voltage in Neurons
Source: Angew Chem Int Ed Engl. 2019 Jan 25;58(8):2341–4. doi: 10.1002/anie.201812967 (PMC6391943; doi:10.1002/anie.201812967)
Supplement: Supplementary file 1 — Supplementary [file ANIE-58-2341-s001.pdf]

Angewandte Chemie
Eine Zeitschrift der Gesellschaft Deutscher Chemiker

Supporting Information

A Chemogenetic Approach for the Optical Monitoring of Voltage in Neurons

Mayya Sundukova, Efthymia Prifti, Annalisa Bucci, Kseniia Kirillova, Joana Serrao, Luc Reymond, Miwa Umebayashi, Ruud Hovius, Howard Riezman, Kai Johnsson, and Paul A. Heppenstall**

anie_201812967_sm_miscellaneous_information.pdf
anie_201812967_sm_Movie_1.avi
anie_201812967_sm_Movie_2.avi
anie_201812967_sm_Movie_3.avi

Reagents and solutions

Salts for solutions were purchased from Sigma-Aldrich, SFP synthase was from NEB.

Plasmids

Plasmids were constructed using standard molecular biology methods. Plasmids used in the study

ER sig – *Bgl*II – protein tag – *EcoR*I – GPi sig
Endoplasmic signal sequence of huCD59 :
ATGGGAATCCAAGGAGGGTCTGTCCTGTTCGGGCTGCTGCTCGTCCTGGCTGTCTTCTGCCATTCAGGTCATA
GC
GPi signal sequence of huCD59 :
CTTGAAAATGGTGGGACATCCTTATCAGAGAAAACAGTTCTTCTGCTGGTGACTCCATTTCTGGCAGCAGCCT
GGAGCCTTCATCCCTAA
The carrier protein of interest is cloned between endoplasmatic signal sequence and GPI signal sequence, the restricition sites *Bgl*II and *EcoR*I :
Sequence of *E. coli* ACP :
ATGAGCACTATCGAAGAACGCGTTAAGAAAATTATCGGCGAACAGCTGGGCGTTAAGCAGGAAGAAGTTACC
AACAATGCTTCTTTCGTTGAAGACCTGGGCGCGGATTCTCTTGACACCGTTGAGCTGGTAATGGCTCTGGAAG
AAGAGTTTGATACTGAGATTCCGGACGAAGAAGCTGAGAAAATCACCACCGTTCAGGCTGCCATTGATTACAT
CAACGGCCACCAGGCGTAA
Sequence of SNAP26f:
GACAAAGACTGCGAAATGAAGCGCACCACCCTGGATAGCCCTCTGGGCAAGCTGGAACTGTCTGGGTGCGAA
CAGGGCCTGCACCGTATCATCTTCCTGGGCAAAGGAACATCTGCCGCCGACGCCGTGGAAGTGCCTGCCCCA
GCCGCCGTGCTGGGCGGACCAGAGCCACTGATGCAGGCCACCGCCTGGCTCAACGCCTACTTTCACCAGCCTG
AGGCCATCGAGGAGTTCCCTGTGCCAGCCCTGCACCACCCAGTGTTCCAGCAGGAGAGCTTTACCCGCCAGGT
GCTGTGGAAACTGCTGAAAGTGGTGAAGTTCGGAGAGGTCATCAGCTACAGCCACCTGGCCGCCCTGGCCGG
CAATCCCGCCGCCACCGCCGCCGTGAAAACCGCCCTGAGCGGAAATCCCGTGCCCATTCTGATCCCCTGCCAC
CGGGTGGTGCAGGGCGACCTGGACGTGGGGGGCTACGAGGGCGGGCTCGCCGTGAAAGAGTGGCTGCTGG
CCCACGAGGGCCACAGACTGGGCAAGCCTGGGCTGGGT

Cell culture and transfection

HEK293T cells (ATCC) were cultured following standard protocols in DMEM medium (supplemented with 10 % FBS, 100 U/ml penicillin/streptomycin) at 37°C, 5% CO_2. Cells were seeded at ~30% confluence on poly-L-lysine (Sigma-Aldrich) coated MatTek glass bottom dishes (MatTek Corporation) and transfected with 100 ng of plasmid DNA and lipofectamine 2000 (Thermo Fisher Scientific) reagent according to the manufacturer's instructions. Cell labeling was performed 24 to 48 hours after transfection.

AAV virus production

Constructs were cloned into an AAV transfer plasmid either with hSyn promoter for SNAP-GPI construct or the CAG promoter for ACP-GPI construct. Recombinant AAV1/2 serotype viral vectors were generated by triple transfection of HEK293T cells and purified on heparin columns as described[1]. Viral genome titers were quantified using qPCR.

Culture of primary dorsal root ganglion neurons and viral induction

[1] McClure, C., Cole, K. L. H., Wulff, P., Klugmann, M. & Murray, A. J. Production and titering of recombinant adeno-associated viral vectors. *J. Vis. Exp.* e3348 (2011).

Dorsal root ganglia (DRG) neurons were isolated from 3-8-week-old mice and enzymatically dissociated as described previously[2] and plated onto glass bottom MatTek dishes coated with poly-L-lysine and laminin. Three to six hours after plating the medium was changed and supplemented with NGF (50ng/ml) and AAV particles, typically at $3x10^{11}$ – $1x10^{12}$ vg/ml. Culture medium was refreshed every day, and neurons were labeled and imaged 3 to 6 days after. AAV1/2 serotype targeted primarily neurons, with negligible targeting of fibroblasts, satellite or Schwann glial cells.

Cell labeling protocol

Cells were washed with serum-free medium immediately before labeling. For labeling with NR12S compound, cells were labeled with 300-500 nM of NR12S for 7 min at room temperature directly in extracellular bath solution. SNAP-derivatized compounds at 0.5-1 µM were incubated in culture medium (DMEM + 10% FBS) for 30 minutes at 37°C. ACP-derivatized compounds at 1-3 µM were incubated at room temperature in serum-free medium with 1 µM of SFP synthase and 10 mM of $MgCl_2$. For control experiments, cells were first pretreated with saturating concentration of CoA (2 mg/ml, with1 µM of SFP synthase and 10 mM of $MgCl_2$) and then labelled with ACP-derivatized compounds as usual. Cells then were imaged directly or after washing with extracellular bath solution.

Epifluorescence imaging of labeled cells

Imaging was performed on Zeiss Axioobserver A1 manual microscope with Axiocam MR and Andor Zyla 4.2P camera. The light source was a 100 W Mercury short arc lamp (OSRAM), and excitation and emission filters for imaging Nile Red compounds were BP 540-552 nm (RFP), BP360-540 nm (Rhodamine) and LP590 nm (RFP), BP570-640 nm (Rhodamine) respectively.

Confocal imaging of live cells and neurons

Confocal images of live cells were performed on a Leica SP5 inverted microscope with resonant scanner using an oil-immersion 40x objective with NA 1.25. An argon laser line 488 nm or solid state 561 nm were used for dye excitation. Emission was collected with a hybrid HyD detector at various wavelengths. Emission spectra were measured and constructed with the in-built lambda-scan mode in the 500-700 nm window, with 5 to 10 nm steps and 5 to 10 nm bandwidth, excitation was performed at 488 nm.

Simultaneous electrophysiology and fluorescence imaging of HEK cells and neurons

Simultaneous optical and electrophysiological recordings were performed on a standard patch clamp setup (HEKA, EPC 10Usb) mounted on the inverted microscope (Zeiss Axioobserver A1). All experiments were performed at room temperature (22°C).

Cells on the microscope stage were perfused with extracellular bath solution consisted of (in mM): 150 NaCl, 5 KCl, 1 $MgCl_2$, 2 $CaCl_2$, 10 HEPES, 5 glucose, pH 7.4 with NaOH, 300 mOsm. Pipettes had resistance of 3–7 mΩ when filled with intracellular solution (in mM): 110 KCl, 2 $MgCl_2$, 0.5 $CaCl_2$, 10 EGTA, 10 HEPES, 3 ATP_2Mg_3, pH 7.2 with KOH, 270 mOsm for neurons and 140 CsCl, 10 HEPES, 10 HEDTA, pH 7.35 adjusted with CsOH, 290 mOsm for HEK293T cells. After establishing whole cell configuration, cell capacitance and access resistance were routinely compensated. All experiments were performed in whole-cell patch clamp configuration. For the recordings of action potentials, switch to current clamp was performed.

---

[2] Stucky, C. L. & Lewin, G. R. Isolectin B(4)-positive and -negative nociceptors are functionally distinct. *J. Neurosci.* **19,** 505 (1999).

Fluorescence excitation was delivered using a 100 W Mercury short arc lamp (OSRAM) through BP 540-552 nm (RFP) filter or BP360-540 nm (Rhodamine) filters. Fluorescence emission was passed through a LP590 nm (RFP) or BP570-640 nm (Rhodamine) filter, and recorded using a Zyla 4.2 Plus (Andor) sCMOS Camera operated with NIS-Elements Ar (Nikon). Voltage sensitivity was found to be similar for the data obtained with these two filters in HEK293T cells, and a Rhodamine filter was used for recordings in DRG neuronal cultures. The field of view was cropped to obtain the desired imaging speed (from 20 fps to 770 fps).

An oil-immersion 40x objective with NA 1.3 was used for imaging HEK293T cells and cultured neurons. Excitation light was delivered at 12 and 28 mW/mm^2 power densities measured with an optical power meter at the imaging plane.

Data analysis

Fluorescence images were analysed in NIS-Elements Ar and ImageJ by manually defining the regions of interest (ROI) and calculating the mean unweighted mean of pixel values within this region. The cell-free region was used for calculating background fluorescence, which was subtracted from the cell fluorescence. When required in the videos, photobleaching was corrected using an exponential decay function.

For analysis of compound fluorogenicity, images of labeled cells without wash were taken, and signal-to-noise ratios were calculated by dividing the background-subtracted mean intensity of the cell membranes by the standard deviation of the background region of interest. Images from several independent labelings were used.

To determine voltage sensitivity, $\%\Delta F/F$ values were generated by subtracting baseline fluorescence at holding potential from fluorescence during the voltage step and plotted against voltage. Voltage sensitivity of different substrates was then compared as $\%\Delta F/F$ per 100 mV depolarization according to the slope of linear fit of the fractional fluorescence change vs membrane voltage or from a -60 mV to +40 mV voltage step. Rise time and decay time of fluorescence response to individual voltage steps were determined by fitting exponential function to the data points. Signal-to-noise ratios for action potentials detected by fluorescence were computed as the ratio of action potential peak amplitude to the standard deviation of fluorescence baseline prior to any depolarization. Exported imaging and electrophysiology data were then analysed using Prism (Graphpad) and custom-written Python scripts. Final data is presented as mean ± SEM if not indicated differently. Values were calculated for multiple action potentials for each neuron, and then averaged, at least three independent cultures and rAAV transductions were used. Statistical tests were performed in SigmaPlot (Systat Software, Inc).

Chemical synthesis

All chemical reagents and anhydrous solvents for synthesis were purchased from commercial suppliers (Sigma-Aldrich, Fluka, Acros) and were used without further purification or distillation. The composition of mixed solvents is given by the volume ratio (v/v). ^1H magnetic resonance (NMR) spectra were recorded on a Bruker DPX 400 (400 MHz) or Bruker AVANCE III 400 Nanobay (400 MHz) with chemical shifts (δ) reported in ppm relative to the solvent residual signals of CD_3OD (3.31 ppm for ^1H) or DMSO-d_6 (2.50 ppm for ^1H). Coupling constants are reported in Hz. High resolution mass spectra (HRMS) were measured on a Micromass Q-TOF Ultima spectrometer with electrospray ionization (ESI) or LTQ Orbitrap ELITE ETD (Thermo fisher). Preparative RP-HPLC was performed on a Dionex system equipped with an UVD 170U UV-Vis detector for product visualization on a Waters SunFire™ Prep C18 OBD™ 5 µm 10×150 mm Column (Buffer A: 0.1% TFA in H_2O Buffer B: acetonitrile. Typical gradient was from 0% to 100% B within 30 min with 4 ml/min flow.)

NR12S was synthesized as described previously[3,4].

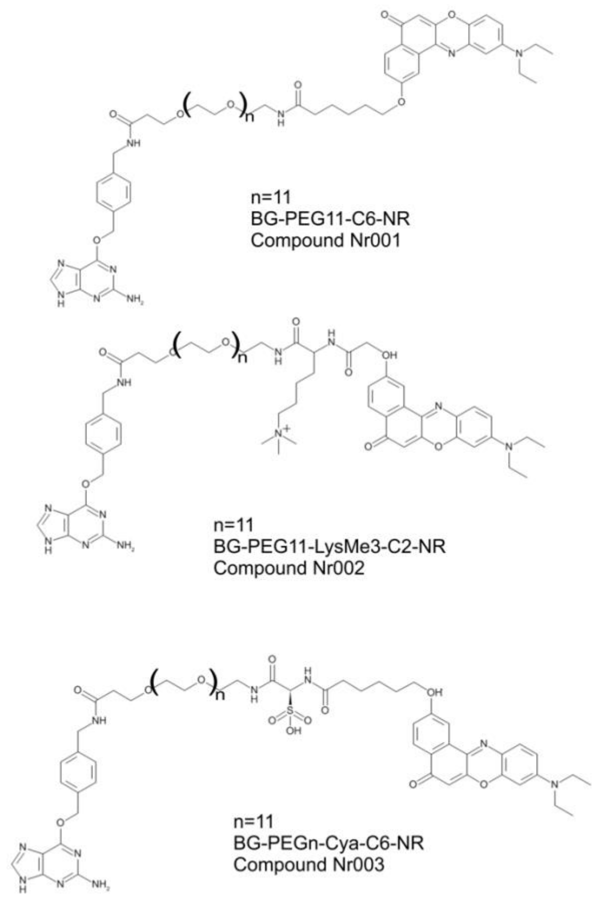

SI Scheme 1. Structures of SNAP-targeted Nile Red derivatives with reactive BG moiety, PEG repeats n=11 and charged groups (no charge, positive and negative) – compounds Nr001, Nr002, Nr003. Compounds were synthesized as described previously[4,5].

CoA-TMR and CoA-ATTO532 were synthesized and purified as described previously[6] using tetramethylrhodamine maleimide (Molecular probes) and Atto532 maleimide (Atto Tec).

---

[3] Kucherak, O. A. *et al.* Switchable nile red-based probe for cholesterol and lipid order at the outer leaflet of biomembranes. *J. Am. Chem. Soc.* **132,** 4907–4916 (2010).

[4] Prifti, E. Fluorescent Probes for Plasma Membrane Proteins Based on Nile Red. *Retrieved from EPFL Infosci.* **7016,** (2016).

[5] Prifti, E. *et al.* A fluorogenic probe for snap-tagged plasma membrane proteins based on the solvatochromic molecule nile red. *ACS Chem. Biol.* **9,** 606–612 (2014).

[6] George, N., Pick, H., Vogel, H., Johnsson, N. & Johnsson, K. Specific labeling of cell surface proteins with chemically diverse compounds. *J. Am. Chem. Soc.* **126,** 8896–8897 (2004).

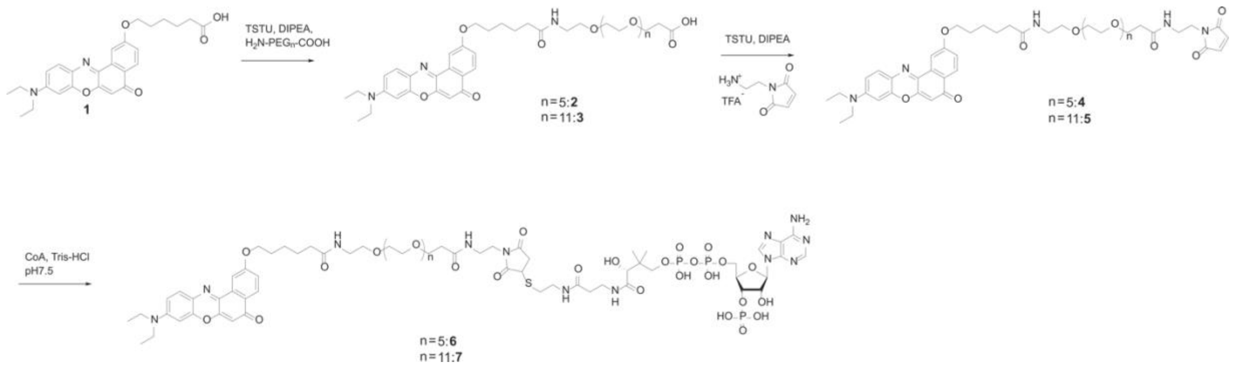

SI Scheme 2. Synthesis of ACP-targeted Nile Red derivatives (CoA-PEGn-Nile Red, n=5, n=11) – compounds **6** and **7**, as described below.

Nile Red-PEG5-COOH 2

Fmoc-PEG5-COOH (6.9 mg, 12 µmol) was dissolved in 0.5 ml MeCN/piperidine 9:1 and the solution was stirred for 20 min at rt. The solvents were evaporated, the redidue was coevaporated 3x with MeCN and dried under high vacuum. Separately, Nile Red-C6-COOH **1**[7] (5.4 mg, 12 µmol) was dissolved in 0.25 ml DMSO, treated with DIPEA (4.1 µl, 24 µmol) and TSTU (3.6 mg, 12 µmol). The solution was incubated for 30 min at r.t. and was transferred in the vial that contained the above deprotected PEG linker. After 1h, the reaction was subjected to RP-HPLC and the product was lyophilized. Yield: 4.7 mg (50%). ^{1}H NMR (400 MHz, MeOD) δ 8.09 (d, 1 H, *J* = 8.8 Hz), 8.00 (d, 1 H, *J* = 2.5 Hz), 7.63 (d, 1 H, *J* = 9.2 Hz), 7.18 (dd, 1 H, *J* = 2.5, 8.8 Hz), 6.92 (dd, 1 H, *J* = 2.6, 9.3 Hz), 6.64 (d, 1 H, *J* = 2.6 Hz), 6.27 (s, 1 H), 4.18 (t, 2 H, *J* = 6.3 Hz), 3.72 (t, 2 H, *J* = 6.3 Hz), 3.66-3.54 (26 H), 3.39 (t, 2 H, *J* = 5.4 Hz), 2.55 (t, 2 H, *J* = 6.3 Hz), 2.30 (t, 2 H, *J* = 7.3 Hz), 1.92 (m, 2 H), 1.77 (m, 2 H), 1.65-1.56 (m, 2 H), 1.30 (t, 6 H, *J* = 6.9 Hz). HRMS (ESI/QTOF) m/z: $[M + Na]^+$ Calcd for $C_{41}H_{57}N_3NaO_{12}^+$ 806.3834; Found 806.3834.

Nile Red-PEG11-COOH 3

Fmoc-PEG11-COOH (10.0 mg, 12 µmol) was dissolved in 0.5 ml MeCN/piperidine 9:1 and the solution was stirred for 20 min at rt. The solvents were evaporated, the redidue was coevaporated 3x with MeCN and dried under high vacuum. Separately, Nile Red-C6-COOH* (5.4 mg, 12 µmol) was dissolved in 0.25 ml DMSO, treated with DIPEA (4.1 µl, 24 µmol) and TSTU (3.6 mg, 12 µmol). The solution was incubated for 30 min at r.t. and was transferred in the vial that contained the above deprotected PEG linker. After 1h, the reaction was subjected to RP-HPLC and the product was lyophilized. Yield: 4.7 mg (37%). ^{1}H NMR (400 MHz, DMSO) δ 8.04 (d, 1 H, *J* = 8.7 Hz), 7.94 (d, 1 H, *J* = 2.4 Hz), 7.88 (t, 1 H, *J* = 5.6 Hz), 7.64 (d, 1 H, *J* = 9.1 Hz), 7.26 (dd, 1 H, *J* = 2.3, 8.7 Hz), 6.83 (dd, 1 H, *J* = 2.4, 9.1 Hz), 6.66 (d, 1 H, *J* = 2.3 Hz), 6.20 (s, 1 H), 4.16 (t, 2 H, *J* = 6.3 Hz), 3.60 (t, 2 H, *J* = 6.3 Hz), 3.52-3.49 (m, 48 H), 3.40 (t, 2 H, *J* = 5.9 Hz), 3.20 (q, 2 H, *J* = 5.6 Hz), 2.44 (t, 2 H, *J* = 6.3 Hz), 2.12 (t, 2 H, *J* = 7.2 Hz), 1.79 (m, 2 H), 1.59 (m, 2 H), 1.45 (m, 2 H), 1.17 (m, 6 H). HRMS (ESI/QTOF) m/z: $[M + Na]^+$ Calcd for $C_{53}H_{81}N_3NaO_{18}^+$ 1070.5407; Found 1070.5405.

Nile Red-PEG5-C2-Maleimide 4

To a 12 mM DMSO solution of Nile Red-PEG5-COOH **2** (200 µl, 2.4 µmol) was treated with DIPEA (1.5 µl, 8.4 µmol) and TSTU (0.87 mg, 2.9 µmol). The solution was incubated for 30 min at r.t. and N-(2-aminoethyl)maleimide trifluoroacetate (0.91 mg, 3.6 µmol) After 1h, the reaction was subjected to RP-HPLC and the product was lyophilized. Yield: 1.6 mg (75%). ^{1}H NMR (400 MHz, MeOD) δ 8.19 (d, 1

[7] Jose, J. & Burgess, K. Benzophenoxazine-based fluorescent dyes for labeling biomolecules. *Tetrahedron* (2006). doi:10.1016/j.tet.2006.08.056

H, *J* = 8.9 Hz), 8.14 (d, 1 H, *J* = 2.5 Hz), 7.77 (d, 1 H, *J* = 9.3 Hz), 7.27 (dd, 1 H, *J* = 8.9, 2.6 Hz), 7.11 (dd, 1 H, *J* = 9.3, 2.6 Hz), 6.81 (m, 3 H), 6.45 (s, 1 H), 4.24 (t, 2 H, *J* = 6.3 Hz), 3.70-3.52 (32 H), 3.40-3.36 (m, 4 H), 2.38-2.35 (m, 2 H), 2.30 (t, 2 H, *J* = 7.3 Hz), 1.97-1.90 (m, 2 H), 1.80-1.73 (m, 2 H), 1.66-1.59 (m, 3 H), 1.32 (m, 6 H). HRMS (ESI/QTOF) m/z: $[M + Na]^+$ Calcd for $C_{47}H_{63}N_5NaO_{13}^+$ 928.4315; Found 928.4316.

Nile Red-PEG11-C2-Maleimide 5

To a 7.5 mM DMSO solution of Nile Red-PEG11-COOH **3** (400 µl, 3.0 µmol) was treated with DIPEA (1.8 µl, 10.5 µmol) and TSTU (1.1 mg, 3.6 µmol). The solution was incubated for 30 min at r.t. and N-(2-aminoethyl)maleimide trifluoroacetate (1.15 mg, 4.5 µmol) After 1h, the reaction was subjected to RP-HPLC and the product was lyophilized. Yield: 2.3 mg (65%). ^{1}H NMR (400 MHz, DMSO) δ 8.05 (d, 1 H, *J* = 8.7 Hz), 7.96-7.94 (m, 2 H), 7.87 (t, 1 H, *J* = 5.5 Hz), 7.65 (d, 1 H, *J* = 9.1 Hz), 7.27 (dd, 1 H, *J* = 2.6, 8.7 Hz), 7.01 (s, 2 H), 6.84 (dd, 1 H, *J* = 2.6, 9.2 Hz), 6.68 (d, 1 H, *J* = 2.6 Hz), 6.21 (s, 1 H), 4.17 (t, 2 H, *J* = 6.3 Hz), 3.56-3.39 (m, 58 H), 3.23-3.18 (m, 4 H), 2.23 (t, 2 H, *J* = 6.6 Hz), 2.13 (t, 2 H, *J* = 7.2 Hz), 1.80 (m, 2 H), 1.60 (m, 2 H), 1.45 (m, 2 H), 1.18 (t, 6 H, *J* = 6.9 Hz). HRMS (ESI/QTOF) m/z: $[M + Na]^+$ Calcd for $C_{59}H_{87}N_5NaO_{19}^+$ 1192.5887; Found 1192.5886.

Nile Red-PEG5-CoA 6

CoA (1.5 mg, 2 µmol) was dissolved in 44 µl 0.1M Tris-HCl pH 7.5 and a 12 mM DMSO solution of Nile Red-PEG5-C2-maleimide **5** (83 µl, 1.0 µmol) was added. After 2h, the reaction was subjected to RP-HPLC and the product was lyophilized. Yield: 0.7 mg (41%). HRMS (nanochip-ESI/LTQ-Orbitrap) m/z: $[M + H2]^{+2}$ Calcd for $C_{68}H_{101}N_{12}O_{29}P_3S^{+2}$ 837.2860; Found 837.2856.

Nile Red-PEG11-CoA 7

CoA (1.5 mg, 2 µmol) was dissolved in 100 µl 0.1M Tris-HCl pH 7.5 and a 5 mM DMSO solution of Nile Red-PEG11-C2-maleimide **6** (200 µl, 1.0 µmol) was added. After 2h, the reaction was subjected to RP-HPLC and the product was lyophilized. Yield: 1.2 mg (60%). MS (ESI) m/z: $[M + Na+H]^{+2}$ Calcd for $C_{68}H_{101}N_{12}O_{29}P_3S^{+2}$ 837.2860; Found 837.2856. HRMS (nanochip-ESI/LTQ-Orbitrap) m/z: $[M + H2]^{+2}$ Calcd for $C_{80}H_{125}N_{12}O_{35}P_3S^{+2}$ 969.3646; Found 969.3644.

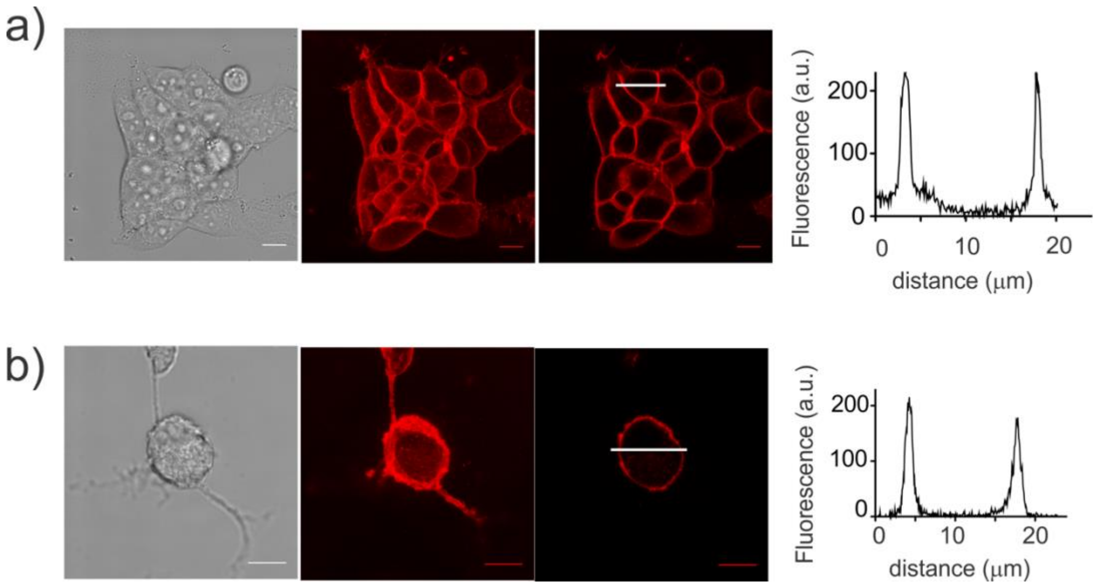

SI Figure 1. NR12S probe labels specifically cell membranes.

a) Representative confocal images of HEK293T cells labeled with 500 nM of NR12S: bright-field image, maximum projection of 46 z-stacks, maximum projection of subset of 11 z-stacks, fluorescence intensity of pixels along the horizontal white line is quantified. b) Representative confocal images of NR12S-labeled cultured DRG neurons: bright-field image, maximum projection of 35 z-stacks, single z-stack image, fluorescence intensity of pixels along the horizontal line is quantified. Stacks acquired at $\Delta z=0.4\mu m$ Scale bar 10 μm.

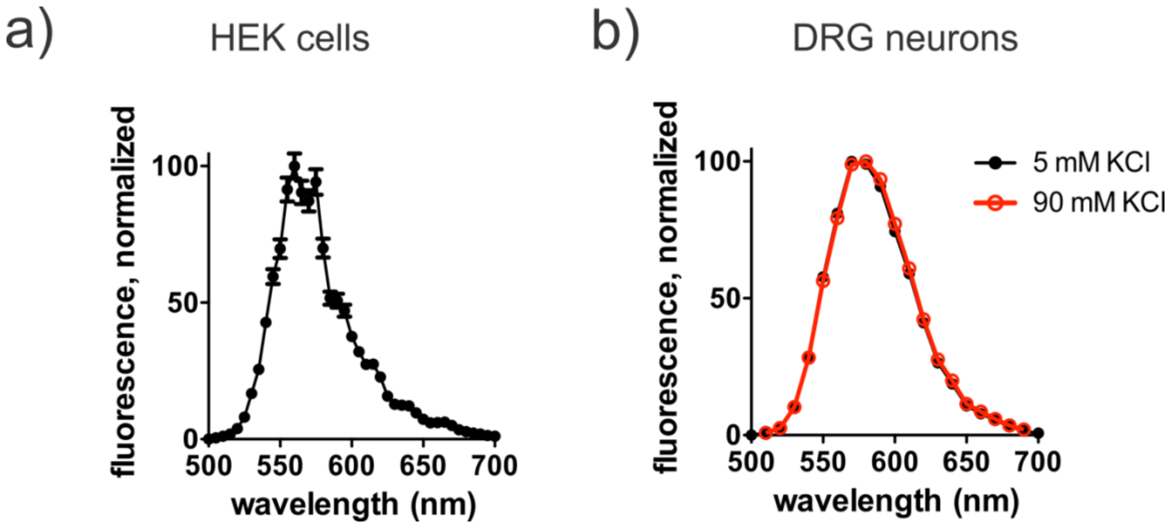

SI Figure 2. Emission spectrum of NR12S measured from live membranes of HEK293T cells (a) and DRG neurons (b) using scanning confocal microscope. Cells were labeled at 500 nM for 7 mins at room temperature. Intensities were normalized to the maximum, mean ± SEM from n=15 cells. Nile Red fluorescence was excited with Argon laser, 488 line, and emission was collected in the 500-700 nm range, with 5 nm steps, 5 nm bandwidth (a) and 10 nm steps, 10 nm bandwidth (b). Upon depolarization with high KCl-containing buffer (90 mM) the emission spectrum did not shift significantly under these experimental conditions.

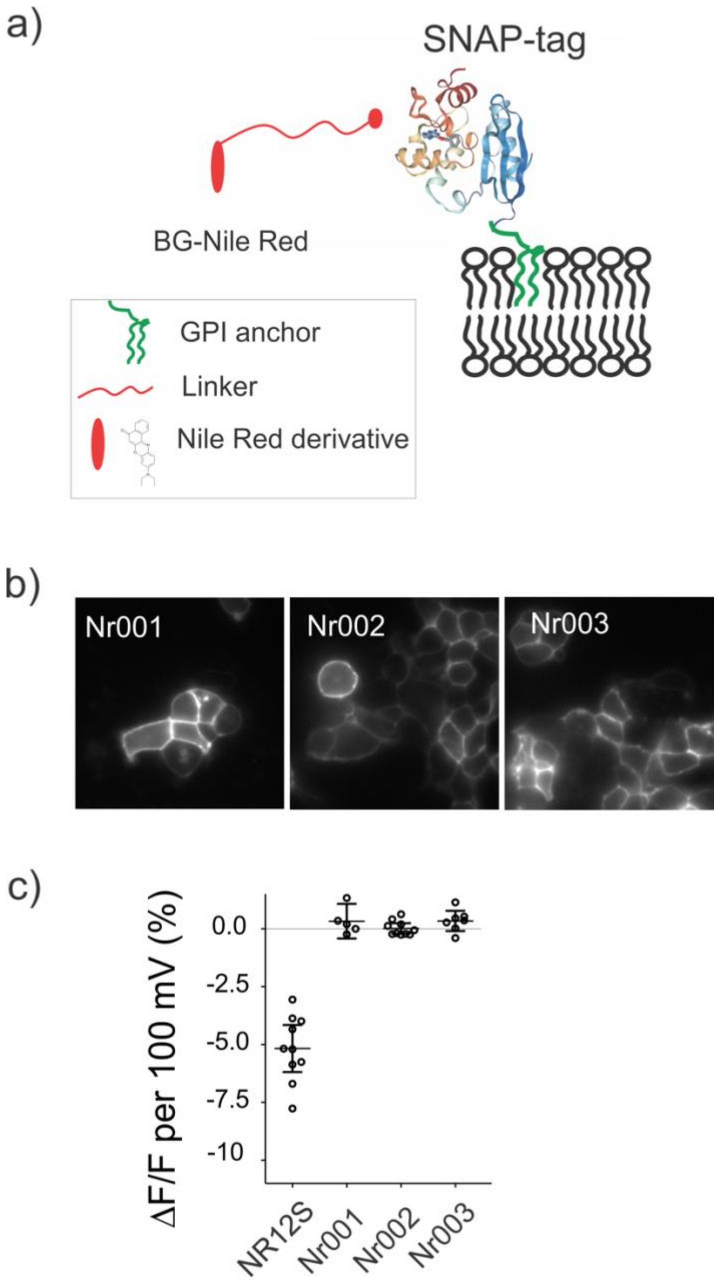

SI Figure 3. Genetic targeting of Nile Red to cells via SNAP-tag.

a) Schematic illustration of tethering Nile Red derivatives with a molecular linker to the 20kDa SNAP-tag. b) Representative wide-field images of HEK293T cells transfected with SNAP-GPI plasmid and labeled with 1 μM of indicated dyes Nr001, Nr002, Nr003, after washes. Scale bar 10 μm. c) Voltage sensitivity expressed as fractional fluorescence change % of tested compounds per 100 mV in patch-clamped HEK293T cells, voltage sensor NR12S is presented as well. Individual points correspond to different cells, line and whiskers represent mean and 95% C.I.

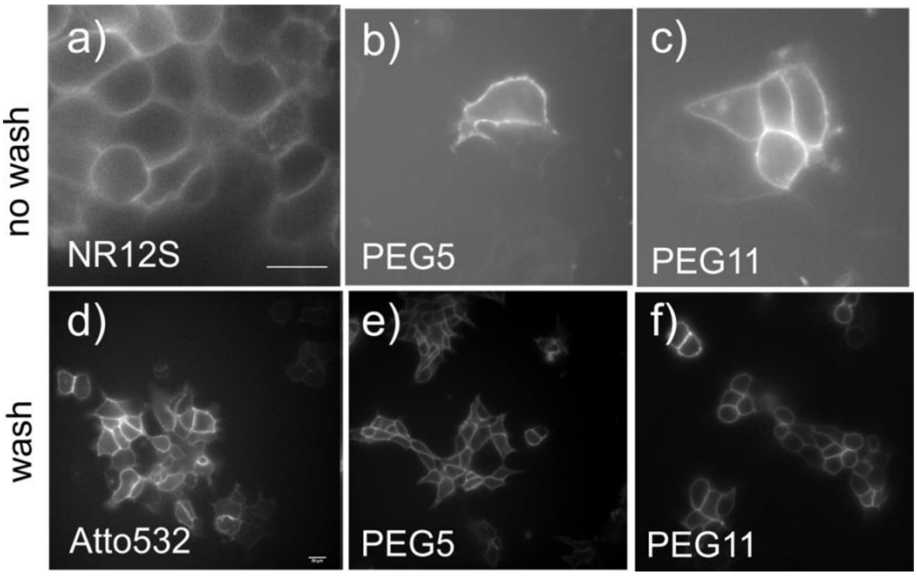

SI Figure 4. ACP-GPI expression and fluorogenic labeling with Nile Red compounds in HEK293T cells.

Representative images of HEK293T cells expressing ACP-GPI and imaged "wash-free" with: a) 500 nM NR12S labeling, b) 2 μM of CoA-PEG$_5$-NR, c) 2 μM of CoA-PEG$_{11}$-NR

Representative images of HEK293T cells expressing ACP-GPI and imaged after 3 washes: d) 1 μM of CoA-Atto532, e) 2 μM of CoA-PEG$_5$-NR, f) 2 μM of CoA-PEG$_{11}$-NR. Scale bar 20 μm.

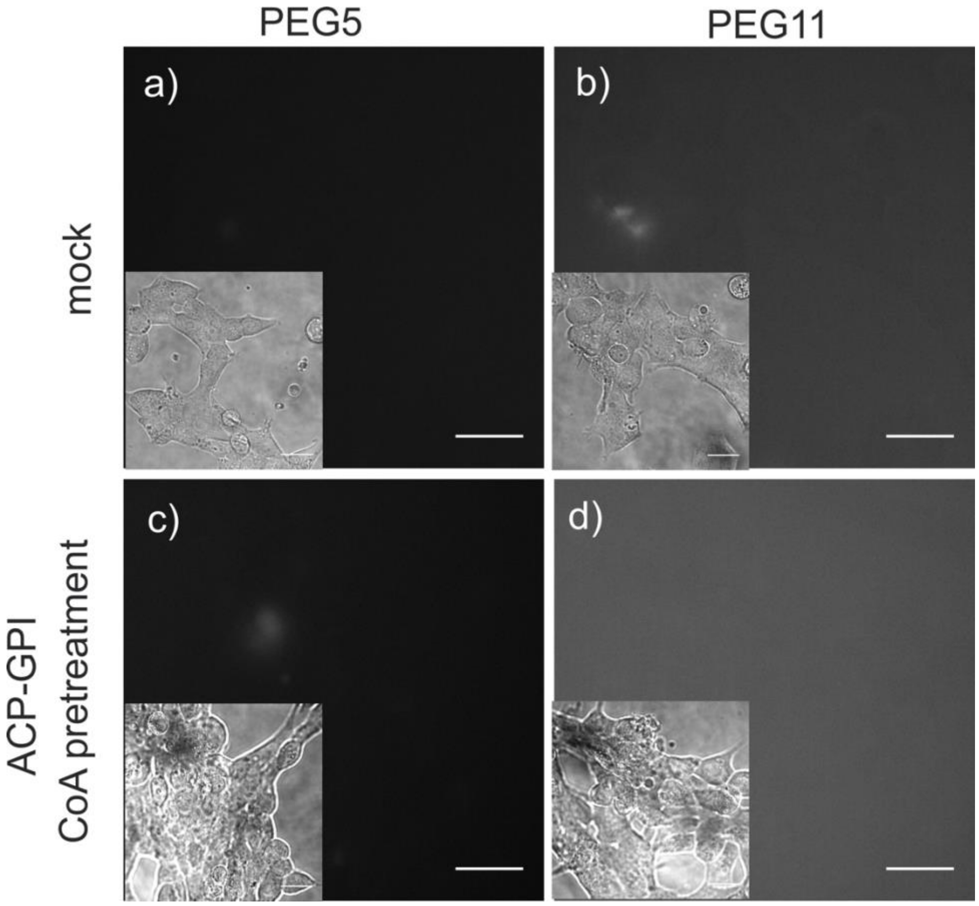

SI Figure 5. Absence of non-specific staining with Nile Red compounds.

Representative images of mock-transfected HEK293T cells imaged "wash-free" with: a) 2 μM of CoA-PEG_5-NR, b) 2 μM of CoA-PEG_{11}-NR. Representative images of HEK293T cells expressing ACP-GPI, pretreatment done with labeling with 2 mg/ml of CoA and afterwards with: c) 2 μM of CoA-PEG_5-NR, d) 2 μM of CoA-PEG_{11}-NR. Imaging was performed "wash-free". Scale bar 20 μm.

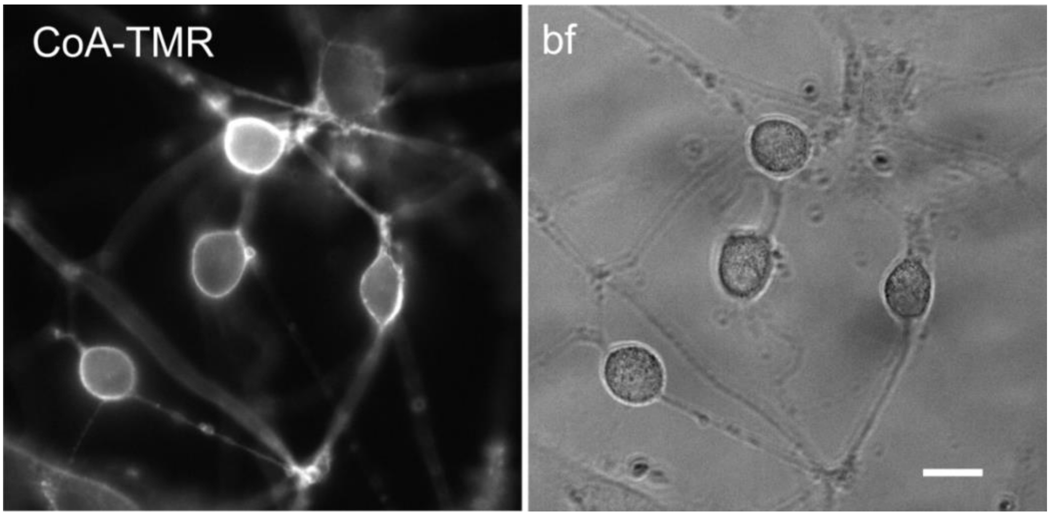

SI Figure 6. ACP-GPI expression in DRG with CoA-TMR. Representative wide-field images of DRG neurons transduced with rAAV1/2 ACP-GPI and labeled with 1 μM CoA-TMR. Distinct membrane staining remained for at least twenty-four hours after labeling. Scale bar 50 μm.

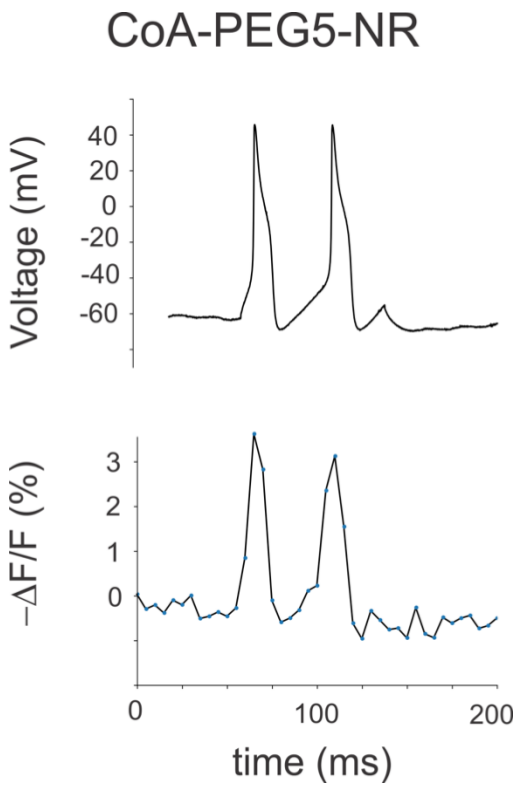

SI Figure 7. Representative single-trial fluorescence (bottom) recordings of current-triggered injection (80 ms) action potentials (voltage trace, top) in DRG neurons with 2 μM CoA-PEG$_5$-NR probe bound to ACP-GPI. Fluorescence images acquired every 3 ms.

name	NR12S	**6**	**7**	Nr001	Nr002	Nr003
substrate	-	CoA	CoA	BG	BG	BG
PEG linker	-	5	11	11	11	11
λ_{max}, nm	581	573	573	632	633	634

SI Table 1. Comparison of fluorescence emission band maxima (λ_{max}) of Nile Red probes targeted to SNAP-tag and ACP-tag. Structures are indicated in SI Scheme 1 and SI Scheme 2. HEK293T cells were transfected with SNAP-GPI and ACP-GPI, spectra were acquired on live cells. Data points were normalized to maximum and fitted with Gaussian to estimate maxima.

Indicator	Fluorophore and mechanism	$\lambda_{exc}/\lambda_{em}$, nm	ΔF/F, % per action potential	SNR in neurons	Light irradiance, mW/mm^2	Linearity of response
STeVI1[a]	Nile Red (solvatochromism and electrochromism)	~530/~590	- 2.2%	13-16	12-28	++
Flare1[1,a]	Rhodopsin with PRIME labeled Cy3 (FRET)	554/568	30% in HEK293[b]	N/A	N/A	+[e]
Voltron[2,a]	Rhodopsin with Halo-tag targeted synthetic dyes (FRET)	525-585/549-609	-23%	N/A	10-23	++
DiO/DPA[3]	DiO dye and dipicrylamine (FRET)	484/501	-16%	7.2	N/A	+[c]
QuasAr-mRuby2[4,a]	archaerhodopsin-voltage sensor and mRuby2 (eFRET)	559/600	-5.4%	7.2	30	+
FlicR1[5,a]	cpmApple and chicken VSD	570/591	2.6%	6	100	+[e]
ASAP1[6,a]	cpGFP and chicken VSD	~490/~520	-4.8%	14.6	8-50	+
Indocyanine green (ICG)[7]	Cyanine derivative	780/818-873	-0.5%[d]	N/A	N/A	++
RhoVR1[8]	Tetramethylrhodamine (photoinduced electron transfer)	565/586	15%	10 - 20	17 - 31	++
ANNINE-6plus[9]	hemicyanine dyes, Stark effect	418/580	20%	N/A	N/A	N/A

SI Table 2. Characteristics of fluorescent GEVI, VSD and hybrid voltage indicators performance tested on neurons. GEVI are color-coded in orange, VSD in magenta, hybrid in blue-green.

Where possible orange/red/far red shifted dyes were chosen. Main groups of indicators are presented, but many are omitted.

Notes: [a] Genetically targeted, [b] Labeling was toxic for neurons, [c] significant capacitative loading, [d] specialized infrared camera needed for detection. [e] Hysteresis in the voltage sensitivity. Linearity expressed as + (linear in small range of membrane voltage), ++ linear over entire physiological range of membrane voltage)

1. Xu, Y. et al. Hybrid Indicators for Fast and Sensitive Voltage Imaging. Angew. Chemie - Int. Ed. (2018).

2. Abdelfattah A.S. et al. Bright and photostable chemigenetic indicators for extended in vivo voltage imaging. bioRxiv 436840; doi: https://doi.org/10.1101/436840 (2018)

3. Bradley, J., Luo, R., Otis, T. S. & DiGregorio, D. A. Submillisecond optical reporting of membrane potential in situ using a neuronal tracer dye. J. Neurosci. 29, 9197–9209 (2009).

4. Zou, P. et al. Bright and fast multicoloured voltage reporters via electrochromic FRET. Nat. Commun. (2014).

5. Abdelfattah, A. S. et al. A Bright and Fast Red Fluorescent Protein Voltage Indicator That Reports Neuronal Activity in Organotypic Brain Slices. J. Neurosci. 36, 2458–2472 (2016).

6. St-Pierre, F. et al. High-fidelity optical reporting of neuronal electrical activity with an ultrafast fluorescent voltage sensor. Nat. Neurosci. 17, 884–889 (2014).

7. Treger, J. S., Priest, M. F., Iezzi, R. & Bezanilla, F. Real-time imaging of electrical signals with an infrared FDA-approved dye. Biophys. J. 107, L09–L012 (2014).

8. Deal, P. E., Kulkarni, R. U., Al-Abdullatif, S. H. & Miller, E. W. Isomerically Pure Tetramethylrhodamine Voltage Reporters. J. Am. Chem. Soc. (2016).

9. Fromherz, P., Hübener, G., Kuhn, B. & Hinner, M. J. ANNINE-6plus, a voltage-sensitive dye with good solubility, strong membrane binding and high sensitivity. Eur. Biophys. J. (2008).

SI Movie Captions.

1. SI Movie 1, to Figure 2c). Fluorescence of STeVI1 (CoA-PEG11-NR) bound to ACP-GPI in HEK293T cell subjected to a 500 ms-long square voltage steps of various magnitudes from holding potential of -60 mV (range -120 mV to +80 mV). Upon depolarization the intensity is decreased, upon hyperpolarization – increased.
2. SI Movie 2, to Figure 3c) *Top,* Pseudocolour computed ΔF/F (%) movie of STeVI1 (CoA-PEG11-NR) bound to ACP-GPI in DRG neuron, firing an action potential upon current injection of 80 pA, 80ms. *Bottom*, greyscale LUT image of the baseline fluorescence from the same neuron. Single-trial recording is presented, with no averaging.
3. SI Movie 3, to Figure 4. Fluorescence of STeVI1 (CoA-PEG11-NR) bound to ACP-GPI in DRG neuron long-term culture, where it tracks spontaneous action potentials (*). Single-trial recording, with no averaging, is presented. Upon depolarization the intensity is decreased, as depicted in the graph below - fluorescence change measured from the neuronal somata membrane.
